# Accumulation of Environmental Radioactivity on the Surface of a High Arctic Ice Cap (Flade Isblink, NE Greenland)

**DOI:** 10.1021/acs.est.3c10755

**Published:** 2024-08-06

**Authors:** Dylan B. Beard, Giovanni Baccolo, Caroline C. Clason, Geoffrey E. Millward, Edyta Łokas, Elena Di Stefano, Sally Rangecroft, Dariusz Sala, Przemysław Wachniew, William H. Blake

**Affiliations:** † School of Geography, Earth and Environmental Sciences, University of Plymouth, Plymouth PL4 8AA, U.K.; ‡ Laboratory of Environmental Chemistry, Paul Scherrer Institut, Villigen 5232, Switzerland; § Oeschger Centre for Climate Change Research, University of Bern, Bern 3012, Switzerland; ∥ Department of Geography, Durham University, Durham DH1 3LE, U.K.; ⊥ Institute of Nuclear Physics Polish Academy of Sciences, Kraków 31342, Poland; # Physics Department, University of Milano-Bicocca, Milano 20126, Italy; ¶ School of Geography, University of Exeter, Exeter EX4 4RJ, U.K.; ∇ Faculty of Physics and Applied Computer Science, AGH University of Krakow, Kraków 30059, Poland

**Keywords:** Arctic, cryoconite, environmental radioactivity, radionuclides, contaminants, ice cap, Greenland

## Abstract

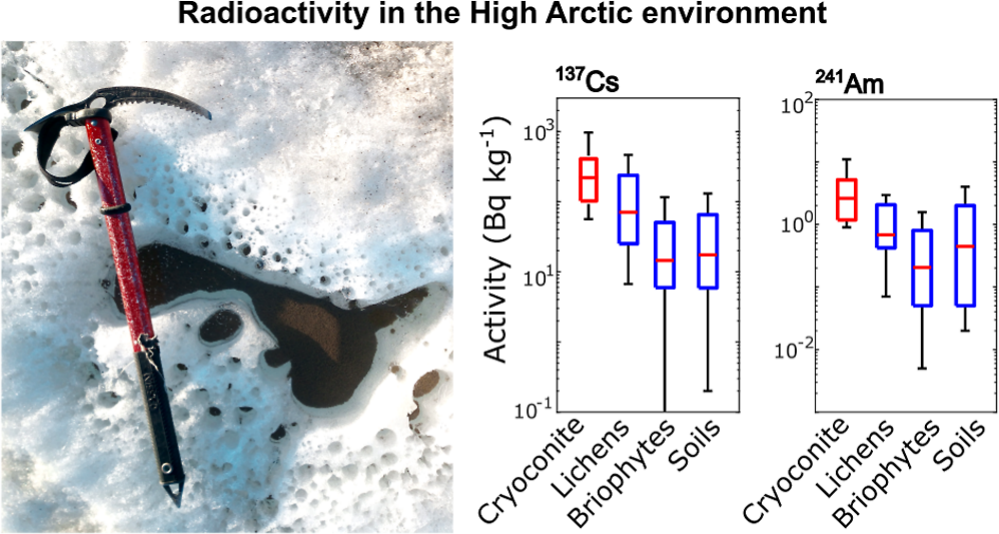

Under climatic warming, glaciers are becoming a secondary source of atmospheric contaminants originally released into the environment decades ago. This phenomenon has been well-documented for glaciers near emission sources. However, less is known about polar ice sheets and ice caps. Radionuclides are one of the contaminants that can be remobilised through ice melting and accumulate in cryoconite material on the surface of glaciers. To understand the cycling of radionuclides in polar glacial contexts, we evaluate the radioactivity of cryoconite samples from Flade Isblink, a High Arctic ice cap in northeast Greenland. The measured radioactivity is among the highest reported across the High Arctic and the highest from Greenland. The high variability observed among the samples is explained by considering the different macroscopic features of single cryoconite deposits. The radioactivity source is compatible with the stratospheric reservoir established during atmospheric nuclear tests and with weapons-grade fissile fuel, likely originating from Novaya Zemlya proving grounds. This study shows that the ability of cryoconite to accumulate radioactivity in remote areas is undisputed, highlighting the need for a deeper understanding of the remobilisation of radioactive species in polar glacial contexts.

## Abstract 2

An Arctic ice cap shows elevated artificial radioactivity on its surface, raising concerns about the secondary release of atmospheric contaminants from glaciers in remote areas under climate change.

## 1 Introduction

The Arctic has received a notable amount of radioactive contamination from distal sources through oceanic currents and atmospheric deposition.^1−3^ Moreover, numerous local activities involving radioactive materials have been carried out in the Arctic, including nuclear weapon tests, dumping of nuclear waste, accidents, and transit of nuclear-powered vessels.^4−6^ The increased exploitation of nuclear energy and radioactive materials in the Arctic is also predicted to intensify in the coming decades,^6,7^ posing new environmental threats.^8,9^ Climate change is affecting the distribution and behavior of contaminants in glaciated environments. Atmospheric warming, which in the Arctic has a rate four times greater than the global average,^10^ is driving the rapid retreat of many glaciers and ice caps.^11^ Glaciers currently act as sinks for atmospheric contaminants.^12^ However, as they retreat and downwaste, glaciers release legacy contaminants previously deposited and stored in snow and ice, transitioning from temporary sinks to secondary sources.^12−15^ Contaminants initially stored in glaciers are increasingly subject to remobilisation and release into proglacial environments.^16−18^ In a rapidly changing cryosphere, understanding the behavior of radionuclides in glacial environments will become increasingly relevant, particularly in the Arctic, where studies remain limited.

Natural and anthropogenic radionuclides of atmospheric origin (fallout radionuclides, FRNs) are known to accumulate on the surface of glaciers within cryoconite deposits.^19,20^ Cryoconite is a material found on glacier surfaces that plays a crucial role in the accumulation of contaminants. It consists of a mixture of mineral and organic matter, with the latter reaching up to 20% of the total mass.^21,22^ The production of extracellular polymeric substances from microorganisms and algae in cryoconite binds mineral and organic matter, increasing its ability to capture airborne materials.^18,23,24^ Furthermore, the interaction between cryoconite and meltwater favors the transfer of contaminants previously stored in snow and ice to the cryoconite itself.^18,19,21,25−27^ FRNs are among the most notable contaminants found in cryoconite on glaciers exposed to fallout from radioactive accidents and atmospheric tests. The levels of radioactivity found in cryoconite can be high, with activities among the highest reported for terrestrial environments,^22^ and often orders of magnitude higher than in other environmental matrices.^20^ Despite the accumulation of radioactivity in cryoconite now being understood to be a global phenomenon,^22^ data from the polar regions is scarce.

The High Arctic (here considered as territories above 70°N) is a region which is simultaneously vulnerable from a radio-ecological perspective while undergoing rapid changes to the cryosphere. Models indicate that the High Arctic is among the most radio-ecologically pristine terrestrial areas of the Northern Hemisphere.^28^ Currently, research on the remobilisation of FRNs and the role of cryoconite is limited in the High Arctic, particularly in the northernmost sectors. To fill these gaps, we report the first analysis of cryoconite radioactivity from a High Arctic glacier: the Flade Isblink ice cap in northeast Greenland. This paper describes the analysis of several radioisotopes in cryoconite from Flade Isblink. We explore the variability in activity concentrations of these radionuclides and assess how macroscopic features of cryoconite influence the accumulation of FRNs. The results show that the radioactivity accumulated in the cryoconite is notable even in remote areas, well above the levels typically observed in the High Arctic. By considering a large number of samples and several FRNs, it was possible to shed light on the accumulation of radioactivity in cryoconite depending on its macroscopic features and tracking the source of artificial radioactivity.

## 2 Materials and Methods

### 2.1 Study Site and Sample Preparation

**Figure 1:**
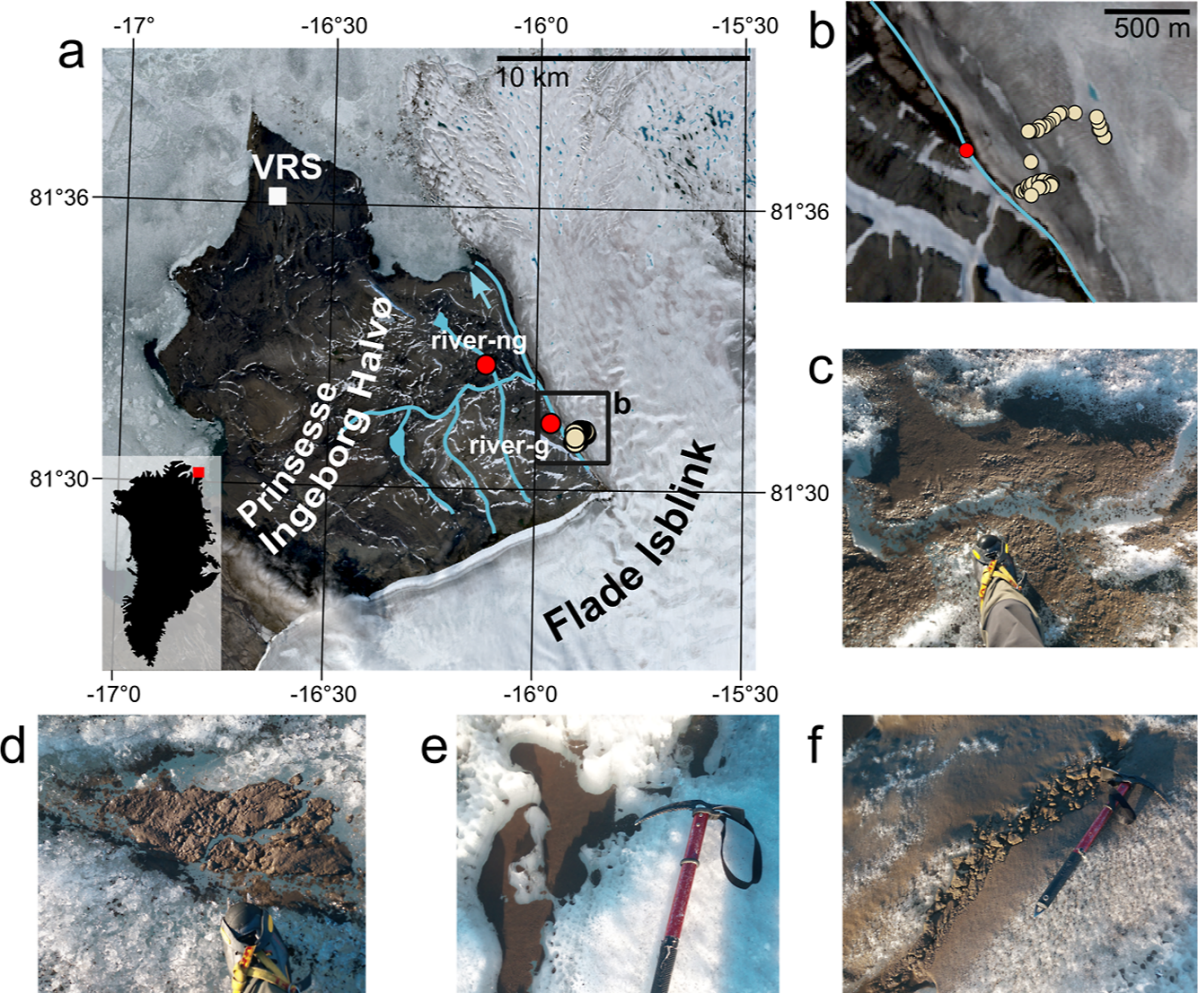
Sampling area and cryoconite subtypes. The study’s geographic context is shown by a satellite image from Sentinel-2 acquired on the 17th of August 2022 (a,b; VRS refers to Villum Research Station). Light circles represent cryoconite sampling sites; red circles are riverine sediment sampling sites, where “river-g″ is the river fed by the glacier and “river-ng” is a river not fed by the glacier. Blue lines highlight the primary hydrological network. Panels (c–f) illustrate a selection of sampled cryoconite deposits: (c) wet granular cryoconite; (d) dry fine cryoconite; (e) wet fine cryoconite; (f) dry granular cryoconite.

Forty-six cryoconite samples were collected on the melting surface of the Flade Isblink ice cap (Greenland, Princess Ingeborg Peninsula, Figure 1) from an area extending 0.25 km^2^ between the first and third of August 2022. Samples were collected to include a range of cryoconite characteristics, including wet/dry and fine/intermediate/granular material (Table S1). Two sediment samples were also collected from rivers in the proglacial area: one from the primary local glacier-fed stream and one from a river not fed by glacier meltwater. After the main Greenland Ice Sheet, Flade Isblink is Greenland’s most extensive body of land ice, covering approximately 8500 km^2^. Since 2010, the ice cap has been subject to increasing melt rates and retreating grounding lines in marine-terminating outlets.^29^ The cryoconite samples were collected in the northern sector of the ice cap, corresponding with one of the outlet glaciers. Further details about sample collection and treatment are presented in the Supporting Information.

### 2.2 Gamma Spectrometry of Radionuclides

FRN activities were analyzed by gamma spectrometry at the Consolidated Radioisotope Facility (ISO 9001 certified) at the University of Plymouth (UK), following an established methodology.^30^ Samples were placed into sealed 4 mL plastic vials and incubated for a minimum of 22 days to allow the establishment of radioactive equilibrium with ^222^Rn. Activity concentrations were determined using a Well detector (ORTEC GWL-170-15 S; N-type) with a counting time of 24 h. Activities of ^214^Pb were subtracted from the total ^210^Pb activity to determine its unsupported component (^210^Pb_exc._). Further details about calibration and quality monitoring are reported in the Supporting Information.

### 2.3 Radiochemical Analysis of Plutonium

The analysis of plutonium isotopes was conducted at the Institute of Nuclear Physics at the Polish Academy of Sciences (Poland) on a subset of 26 samples using an established methodology.^19,31,32^ Samples were first mineralized through acid digestion, and ^242^Pu was used as an internal tracer. The separation of Pu took place using the Dowex-1 exchange resin. After separation, Pu was precipitated on membranes and measured through Alpha Analyst 7200 spectrometer (Mirion Technologies) with a PIPS detector presenting an active surface of 450 mm^2^. The ^240^Pu/^239^Pu atomic mass ratio was determined with inductively coupled plasma–mass spectrometry (ICP–MS).^17^ Membrane filters were dissolved with concentrated acids. U and Th traces were removed from dissolved samples with exchange resins. Purified samples were then measured through the Agilent 8900#100 ICP–MS/MS (Agilent Technologies) coupled with an Aridus desolvating nebulizer. The method is described further in the Supporting Information.

### 2.4 Radioactivity Data Treatment

All activity and MDA (minimum detectable activity) values refer to dry mass. FRN activities of single samples are reported with their 2-sigma counting error and were decay-corrected to the date they were collected. Data below MDA were treated as MDA/2 for the purposes of this study. Two algorithms were applied to analyze the correlation among the considered variables: multidimensional scaling and hierarchical clustering. Further details are provided in the Supporting Information.

### 2.5 Grain Size Analysis

Granulometric analyses were conducted on a subset of 10 samples considering all the collected cryoconite types (wet/dry, fine/intermediate/granular). Analyses were performed through the Coulter counter technique, a reference for the analysis of fine mineral particles.^33^ A Beckman Multisizer 4 equipped with a 100 μm orifice was used to measure particles between 2 and 60 μm, divided into 400 size channels. Samples were dried and gently worked with a mortar to break the cryoconite granules. An aliquot of about 0.5 g was added into a clean electrolyte solution and agitated until measurement. Five runs were performed for each sample, and 0.5 mL was analyzed for each run. Precautions were taken to limit the settling of larger particles during the analysis. Further details are provided in the Supporting Information.

## 3 Results and Discussion

Cryoconite was abundant on Flade Isblink, with deposits at the bottom of water-filled holes and in surface deposits poorly connected to the supraglacial hydrologic network (Figure 1). Samples were grouped according to the aggregation state of the material (fine, intermediate, or granular) and the degree of interaction between deposits and meltwater (wet for cryoconite accumulated at the bottom of water-filled cavities in the ice or at the bottom of supraglacial meltwater channels; dry for cryoconite not in contact with meltwater) (Table S1). The particle size of the samples was well-described by a Gaussian distribution presenting a mode spanning between 13.9 and 26.7 μm. No significative differences in particle size were found between the cryoconite classes (Figure S4).

### 3.1 Radioactivity of Cryoconite on Flade Isblink

#### 3.1.1 Artificial FRNs

**Figure 2:**
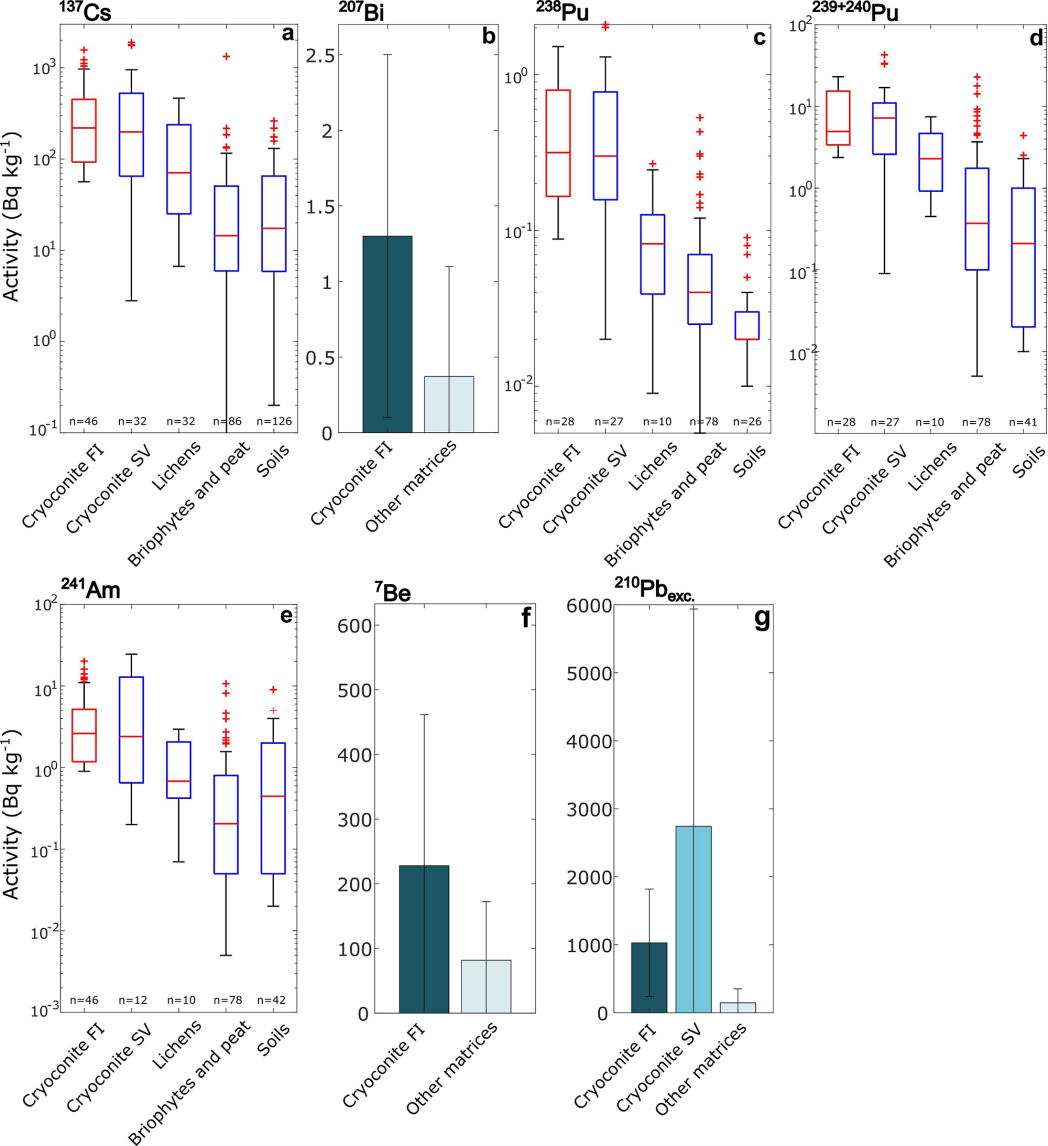
. FRN activities in Flade Isblink cryoconite compared to published activities in other samples. Samples (cryoconite FI) are compared to matrices from Greenland and the terrestrial High Arctic (lat ≥ 70°N), including Svalbard cryoconite (Cryoconite SV), lichens, bryophytes/peat and soils.^32,34−44^ Box-Whiskers plots contain data for all FRNs except ^207^Bi, ^7^Be and ^210^Pb_exc._. For the latter, bar charts show the mean value for cryoconite compared to available data from the Arctic without distinguishing the sample type. For the bar charts, error bars correspond to one standard deviation.

The following artificial FRNs were detected in Flade Isblink cryoconite: ^137^Cs, ^207^Bi, ^241^Am, ^238^Pu and ^239+240^Pu. Their median (reported here rather than the mean because of the high variability observed across the samples) activities are 220, 0.9, 2.6, 0.3, and 4.9 Bq kg^–1^, respectively (Figure 2 and Supporting Information), while maximum activities detected in single samples are 1600 ± 200, 6.4 ± 2.5, 20 ± 3, 1.5 ± 0.2 and 23.1 ± 1.7 Bq kg^–1^ (±*n* refers to experimental uncertainty of single measurements). To provide context for these values, for ^137^Cs, Pu isotopes, and ^241^Am, the radioactivity of Flade Isblink cryoconite was compared with other environmental matrices monitored in the terrestrial High Arctic, including lichens,^34−37^ bryophytes,^35,36,38^ soils^32,34,35,39−44^ and cryoconite from glaciers on Svalbard^19,32^ (Figure 2a,c,d,e). Data from sites where nuclear tests occurred or that were subject to severe fallout from Chernobyl were not considered. The median FRN activities of cryoconite from Flade Isblink are the highest among all matrices for the considered artificial FRNs, except ^239,240^Pu, for which a higher median value (7.2 vs 4.9 Bq kg^–1^)^19^ was found in cryoconite from Svalbard.^19,32^ The highest activities in cryoconite from Flade Isblink are for ^137^Cs, a high-yield product from ^235^U and ^239^Pu fission and a dominant constituent of nuclear fallout.^45^ The median activity of ^137^Cs in Flade Isblink cryoconite is three times the median value of Arctic and Greenlandic lichens (220 vs 70 Bq kg^–1^) and 15 times the value found in Arctic bryophytes. Only cryoconite from Svalbard contains ^137^Cs contamination of a comparable level (median activity 200 Bq kg^–1^).

A similar picture is seen with Pu and ^241^Am, albeit with lower absolute activities. These artificial radionuclides were injected into the stratosphere as a consequence of nuclear atmospheric tests, enabling their transport around the globe.^46^^238^Pu has a different primary source: the re-entry of the SNAP-9A satellite occurred in 1964, which resulted in ^238^Pu reaching the Arctic via mesospheric transport.^47^ The ratio between the median concentrations of FRNs in cryoconite from Flade Isblink and those for lichens, bryophytes/peat and soils is 4, 12, and 5 for ^241^Am; 4, 8, and 18 for ^238^Pu; and 1, 12, and 20 for ^239,240^Pu. Another artificial FRN observed in cryoconite, ^207^Bi (Figure 2b), is only sporadically considered in radio-ecological studies since its environmental concentration is extremely low.^48^ Its production and release into the environment have been related to specific high-yield thermonuclear tests, such as those carried out in Novaya Zemlya or in the Pacific proving grounds.^49^ One of the few studies about its occurrence in the Northern Hemisphere, and the only research within the Arctic, focused on Greenland, where this radionuclide was found in the 1980s.^36^^207^Bi was detected in six cryoconite samples from Flade Isblink, with activity concentrations among the highest reported globally^48^ (6.4 ± 2.5 Bq kg^–1^ is the highest measured activity).

#### 3.1.2 Natural FRNs

Two natural FRNs were also detected in cryoconite from Flade Isblink, with concentrations higher than the typical environmental background: ^7^Be and ^210^Pb_exc_ (Figure 2f,g). ^7^Be is a short-lived (*t*_1/2_ = 53.2 d) cosmogenic product,^50^ while ^210^Pb_exc._ is a decay product of ^222^Rn, a gaseous geogenic radionuclide belonging to the ^238^U decay chain.^51^ As for artificial FRNs, the activity concentrations of these natural FRNs are higher than seen in previous data from Greenland and the terrestrial High Arctic. Given data scarcity in these regions, conducting a detailed comparison was not possible. However, a comparison with available data shows that the activities of these FRNs in cryoconite are above the background observed in the High Arctic. For ^210^Pb_exc._, only cryoconite from Svalbard contained comparable activities^19,32^ (Figure 2f,g).

#### 3.1.3 Geogenic Radionuclides

**Figure 3:**
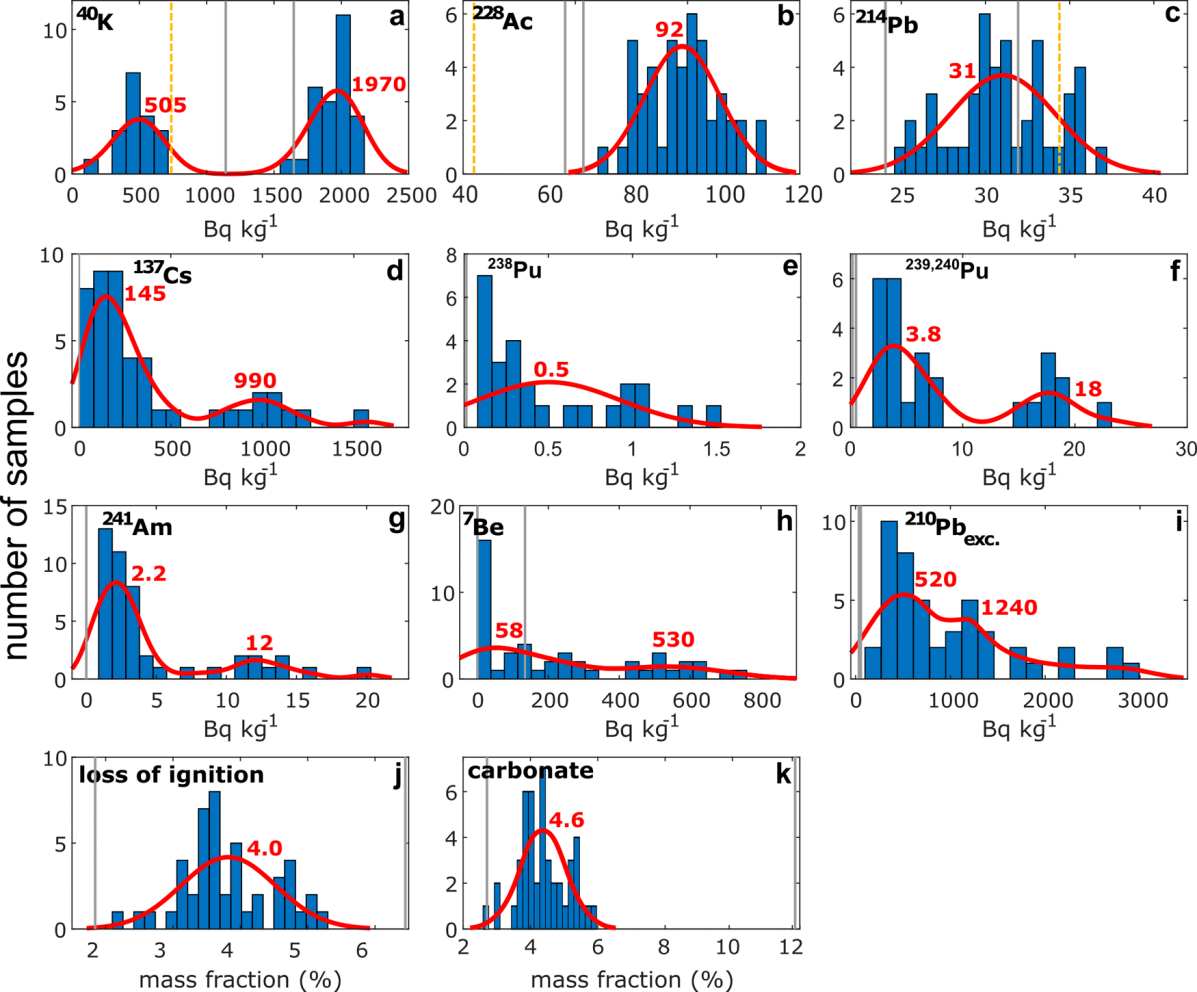
Frequency distributions of radionuclides and key geochemical data for cryoconite on Flade Isblink. Red curves are distribution fits applied to the original data, and red numbers show peak values. Normal (panels b,c,e,j,k) or kernel (panels a,d,f,g,h,i) fits were used. See Figure S1 for an evaluation of data normality. The yellow dotted line in panels a–c refers to the average crustal activity of geogenic radionuclides.^52^ Gray lines represent the two local riverine sediment samples. In most samples, ^207^Bi was below the minimum detectable amount and was thus omitted.

Radiometric analyses revealed the occurrence of geogenic radionuclides in cryoconite: ^214^Pb, ^228^Ac and ^40^K. The first two belong to the decay chains of ^238^U and ^232^Th, and the latter is a primordial radioactive nuclide. Figure 3a–c shows the activity distribution of ^214^Pb and ^228^Ac, where data was fitted with a normal function (see Figure S1 for further information on data normality). The normal distributions of ^228^Ac and ^214^Pb activities peak at 92 and 31 Bq kg^–1^, respectively. For ^214^Pb, this corresponds to the Upper Continental Crust^52^ (UCC) reference value for the ^238^U chain (33 Bq kg^–1^). For ^232^Th, there is a deviation (UCC reference 43 Bq kg^–1^; cryoconite distribution peak 92 Bq kg^–1^), but it still lies within the UCC natural variability.^53^ The data for ^40^K does not show a normal distribution and is characterized by two clusters with activities of 500 and 2000 Bq kg^–1^. The activity of the first cluster is close to the UCC reference (710 Bq kg^–1^), while the second has an activity almost three times higher (1970 Bq kg^–1^). Since samples were collected a few kilometres from the ocean, the abundance of potassium in the second cluster could be related to the influence of sea spray.^54^

#### 3.1.4 Correlation Analysis

**Figure 4:**
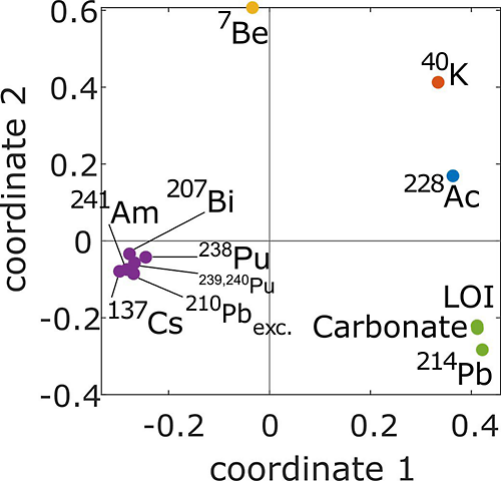
Pearson’s correlation matrix represented as a scatter plot through multidimensional scaling. Variables were clustered using a classic hierarchical algorithm as a function of their respective distance. Clusters are illustrated with shared colors.

The degree of correlation and clustering between the radionuclides, loss of ignition (LOI) and carbonate content was explored by applying multidimensional scaling and hierarchical clustering to a Pearson’s correlation matrix (Table S3). Results are illustrated in Figure 4, where the closer the variables are to one another, the higher the degree of correlation. The most evident cluster contains all FRNs except for ^7^Be. It includes both natural and artificial FRNs (^207^Bi, ^210^Pb_exc._, ^137^Cs, ^238^Pu, ^239,240^Pu, ^241^Am), which implies that the correlation between these FRNs does not reflect a common source but is more likely a common accumulation mechanism.

^7^Be does not correlate with the other FRNs, likely because this cosmogenic nuclide is present only in fresh snow and precipitation, not ice, due to its short half-life. As already observed in the Alps, the presence of ^7^Be in cryoconite is related to recent exchanges between meltwater and cryoconite that occurred in the season when sampling took place.^25^ Geogenic radionuclides are also not clustered despite the first multidimensional scaling coordinate grouping them within the positive domain. ^40^K is the most isolated variable, which may be explained by its delivery to the surface of Flade Isblink through marine aerosols. ^228^Ac and ^214^Pb are classified within two separate clusters, likely because of their fractionation in different mineral phases. ^214^Pb correlates well with LOI and carbonate content. The organic content of cryoconite, estimated by proxy through LOI, typically increases with age,^55^ while carbonate can precipitate in cryoconite during winter freezing.^56^ Cryoconite surviving multiple summer/winter cycles can thus accumulate carbonate and organic matter, explaining their correlation. The presence of ^214^Pb in this cluster supports that cryoconite with higher organic matter and carbonate contents better accumulates uranium-bearing mineral particles.^57^

### 3.2 Influence of Cryoconite Characteristics on the Accumulation of Radioactivity

Distributions reported in Figure 3 show that the activities of FRNs in cryoconite are highly variable. This is also confirmed by high coefficients of variation (CV %). ^241^Am, ^7^Be and ^137^Cs have the highest variability, with a CV % of 103, 102, and 102, respectively. The variability of ^207^Bi, ^238^Pu, ^239+2420^Pu, and ^210^Pb_exc._, is also high, with a CV % between 75 and 90%. Geogenic radionuclides (except ^40^K), LOI, and carbonate content have a low CV % (≤20%), revealing a homogeneous composition that is well-fitted by a Gaussian function. ^40^K has a CV % of 53%. Figure 3 shows that most FRNs and ^40^K display two modes, pointing to the presence of two clusters in the data. The high variability of FRN activities is commonly attributed to heterogeneous sources and depositional fluxes. This is not likely, as the samples were collected from an area of only 0.25 km^2^.

**Figure 5:**
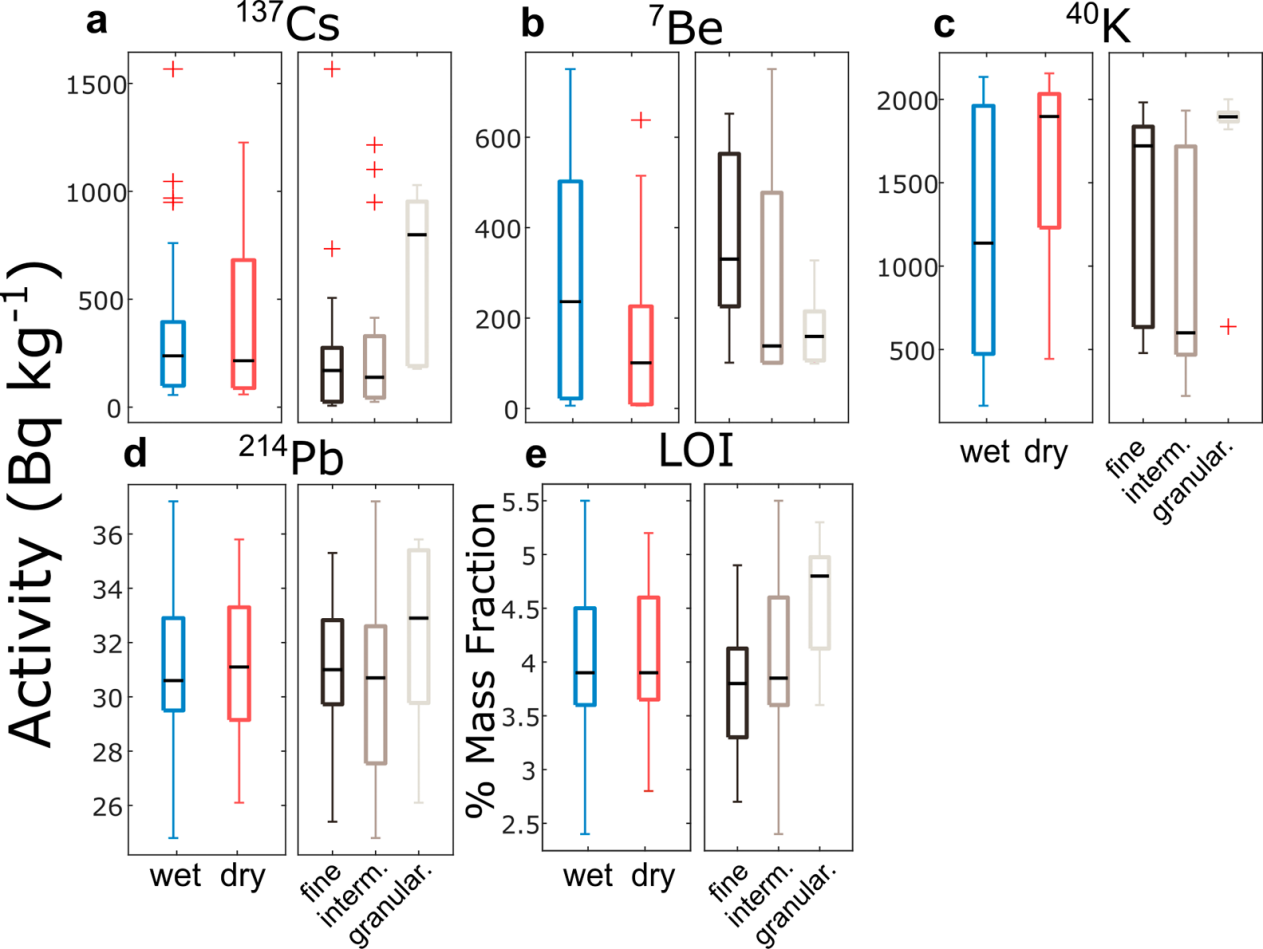
Activity concentrations of cryoconite based on determined characteristics. Activity concentrations in cryoconite are grouped according to the degree of interaction with meltwater (wet and dry) and the aggregation state (fine, intermediate, granular; panels a–d). The same scheme is adopted for LOI (panel e). Only selected nuclides are shown; complete data are reported in Table S2 and Figure S2.

Flade Isblink’s ice surface was covered in both granular and fine cryoconite material. This dual nature of cryoconite is well-known.^21^ Well-developed cryoconite granules have been linked to developed microbial activity.^26,57−60^ In contrast, poorly developed or absent granules have been related to low microbial activity, typical of immature cryoconite.^25,58^ Cryoconite granules repeatedly grow and disintegrate over multiple years on glaciers.^58^ In our samples, the activities of all FRNs, except for ^7^Be, are higher in granular cryoconite than in fine cryoconite (Figure 5a). Considering ^137^Cs as representative of long-lived radionuclides, its median activity in fine and granular cryoconite is 214 Bq kg^–1^ and 820 Bq kg^–1^, respectively. FRNs enriched within granular cryoconite (^137^Cs, ^210^P_exc._, plutonium isotopes, ^241^Am) have half-lives exceeding decades and were likely accumulated across several melt seasons. This indicates a long accumulation history in granular samples, which agrees with their maturity. ^7^Be is the only FRN depleted in granular cryoconite compared to fine samples (median values of 75 vs 270 Bq kg^–1^, Figure 5b). The scarcity of short-lived ^7^Be in granular cryoconite suggests a less intense biogeochemical exchange activity prior to sampling. Furthermore, granular cryoconite has a smaller specific surface area than fine cryoconite, slowing absorption processes in the short term. Conversely, fine cryoconite is enriched in ^7^Be and depleted in long-lived FRNs, indicating high exchange activity prior to sampling and that this kind of cryoconite formation is relatively recent. Geogenic nuclides display only moderate differences depending on their aggregation state (Figure 5d).

Considering wet and dry cryoconite, ^7^Be and ^40^K are the nuclides exhibiting the most significant differences (Figure 5b, c). Wet cryoconite is richer in ^7^Be than dry samples (median values of 240 and 100 Bq kg^–1^, respectively). This is expected, as the short half-life of ^7^Be implies that only cryoconite that was in contact with meltwater at or near the time of sampling could accumulate activity from meltwater. ^40^K behaves oppositely, with higher mean activities in dry samples (1900 Bq kg^–1^) than in wet samples (1140 Bq kg^–1^). This can be attributed to the leaching of soluble elements such as potassium within meltwater, aligning with previous findings.^27^ The elevated ^40^K activity in dry cryoconite samples may be attributed to the influence of marine aerosols, which, once deposited, are retained by cryoconite if meltwater is scarce. The interaction with meltwater does not significantly control LOI and carbonate content (Figures 5e and S2). However, there is some variability in LOI and carbonate content in relation to the aggregation state. Granular cryoconite is richer than fine cryoconite in both organic matter (4.9 vs 3.8%) and carbonates (5.4 vs 4.5%). High carbonate percentages have been linked to the degree of maturity of cryoconite, as carbonate precipitates in cryoconite during winter freezing.^56^ Organic matter has also been correlated with the degree of maturity of cryoconite.^26^ The enrichment of carbonate, organic matter and long-lived FRNs in granular cryoconite is thus consistent with the supposed maturity of this material. According to granulometric analyses (Figure S4), the size of mineral particles in cryoconite from Flade Isblink does not depend on wet/dry and fine/granular features. Samples present a particle size mode ranging from 13.9 to 26.7 μm, regardless of the considered cryoconite type.

### 3.3 Comparison Between Cryoconite and Local Riverine Sediments

Two samples were collected from local river sediments to assess the radioactivity of cryoconite in relation to the Prinsesse Ingeborg Halvø background, one from a river fed by glacial meltwater (river-g), and one from a nonglacially fed river (river-ng). The nonglacially fed river drains a significant portion of the deglaciated area of Prinsesse Ingeborg Halvø, while the other is fed by Flade Isblink meltwater and is highly dynamic from a sedimentological point of view. The river-g sample was collected from a bar, providing a reference to evaluate how FRNs behave in glacial sediments spread through the proglacial environment. While only two samples are considered here due to constraints on sampling time in the field, the sediments are regarded as well-mixed and representative of the catchments within which they were retrieved.

The activity of FRNs in riverine sediments is orders of magnitude lower than that observed in cryoconite, while geogenic nuclides are present with typical crustal concentrations (see Table S2 for complete data). The ratios between the mean activities of ^137^Cs, ^239,240^Pu, and ^210^Pb_exc._ in cryoconite and riverine sediments are 104, 50, and 42, respectively, while enrichment is less evident for ^7^Be (ratio of 4.5). Moreover, ^7^Be is the only FRN exhibiting a significantly different concentration between the two riverine samples (below MDA and 90 ± 30 Bq kg^–1^ in river-g and river-ng, respectively). This difference can be attributed to different water sources: the nonglacial river is fed by meltwater from recent snow thawing, explaining the presence of short-lived ^7^Be, while the glacial stream is fed by meltwater from Flade Isblink, primarily produced by melting of old ice where ^7^Be has decayed.

### 3.4 Sources of Radioactivity in the High Arctic

**Figure 6:**
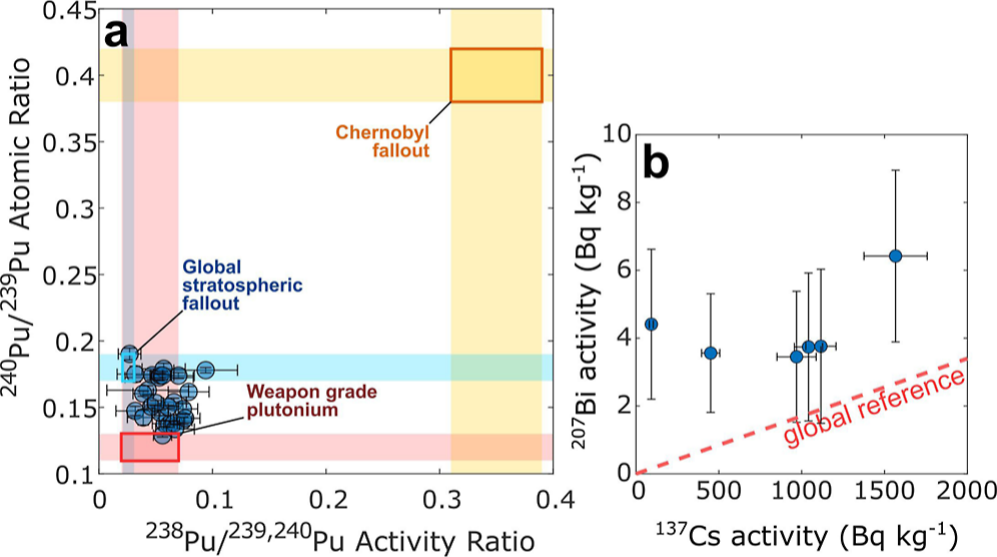
Atomic ratios of Pu isotopes and ^137^Cs vs ^207^Bi activities in Flade Isblink cryoconite. Panel a shows ^240^Pu/^239^Pu vs ^238^Pu/^239,240^Pu ratios, and panel b shows ^207^Bi vs ^137^Cs activities. Reference values are taken from literature^47,48,66−69^ and all data is decay-corrected to August 2022.

Plutonium isotopes reveal that the signature of artificial radioactivity in cryoconite is consistent with a mix of fallout originating from the global stratospheric reservoir and weapons-grade plutonium (Figure 6a). The reference ^240^Pu/^239^Pu ratio for global fallout is 0.18,^61^ while Flade Isblink cryoconite exhibits a mean value of 0.15 with a standard deviation of 0.02. Such values point to an excess of ^239^Pu, the primary fissile fuel used in nuclear and thermonuclear weapons. This finding agrees with the geographic position of northern Greenland, which is within ∼1800 km westward of Novaya Zemlya, a primary USSR nuclear weapon-proving ground. However, air masses in the High Arctic often travel counterclockwise around the North Pole, increasing the relative distance FRNs would have to travel from Novaya Zemlya to Flade Isblink.^62^ It is known that radioactive fallout from USSR devices, including those tested in Novaya Zemlya, is characterized by a lower ratio compared to both global and US test-related fallout.^63,64^ The fallout from Chernobyl does not play a significant role, confirming the limited influence of this event in northeast Greenland.^65^

A further confirmation that USSR tests played a dominant role comes from ^207^Bi, whose diffusion in the Northern Hemisphere was related to specific high-yield tests conducted in Novaya Zemlya.^36^ Despite the limited number of samples where ^207^Bi was quantified (6 of 46), the ^207^Bi/^137^Cs ratio (Figure 6b) reveals that the samples where this rare FRN was detected present an excess of ^207^Bi compared to ^137^Cs. On average, the ^207^Bi content is seven times higher than expected from global stratospheric fallout. These results further support the hypothesis that NE Greenland was exposed to the fallout produced by Soviet tests in Novaya Zemlya.

### 3.5 Summary and Perspectives

This study addresses the regional gap in understanding FRNs in High Arctic glacial settings. Prior to this study, there was minimal information about radioactive contamination in northern Greenland. Previous model results had identified this region as one of the most radio-ecologically pristine areas in the Northern Hemisphere, but no direct data existed to support this.^28^ However, our findings reveal that cryoconite accumulates FRNs even in remote areas like Flade Isblink. The radioactive concentrations in cryoconite collected from this ice cap are some of the highest recorded across the High Arctic region, with artificial ^137^Cs and natural ^210^Pb_exc._ exceeding 1000 Bq kg^–1^ in several samples. Furthermore, rare artificial radionuclides such as plutonium isotopes, ^241^Am, and ^207^Bi were detected at concentrations orders of magnitude higher than the typical Arctic environmental background. Mineral particles in cryoconite samples are extremely fine, with the most abundant particles having a size between 14 and 27 μm. The presence of fine particles partially explains the ability of cryoconite to accumulate radionuclides, as it is known that FRNs accumulate preferentially on such particles.^70^ The dominance of fine particles can likely be attributed to two factors: glacial activity, which comminutes rock fragments into fine debris, and long-range aeolian transport, which deposits micrometric dust particles on glacier surfaces.^71^ This dominance could be a relevant factor in explaining, at least partially, why cryoconite on glaciers worldwide is enriched with FRNs.^22,71^

Fine cryoconite is depleted in long-lived FRNs but is rich in ^7^Be, whose half-life is 53 days. Fine cryoconite is interpreted as a young and biogeochemically active sediment. It efficiently accumulates short-lived ^7^Be, but the short accumulation history prevents it from building up a significant burden of long-lived FRNs. Granular cryoconite behaves oppositely, containing high concentrations of long-lived species while being depleted in ^7^Be. Low ^7^Be activities suggest that granular cryoconite was not efficiently absorbing radioactivity prior to sampling. However, the burden of long-lived FRNs points toward a prolonged accumulation history. We interpret granular cryoconite as relatively old and inactive, likely because binding sites are already near saturation. The higher concentration of organic matter and carbonates in granular samples, known to accumulate in cryoconite over time,^26,56^ also supports this interpretation. Age and the physical state of cryoconite appear relevant for explaining the intraglacier distribution of radioactivity. It is recommended that future studies consider an adequate number of samples, considering the different macroscopic features of cryoconite deposits, in particular, the aggregation state and the degree of interaction with the supraglacial hydrological network.

Besides being notable in terms of absolute activities, the concentrations of FRNs in cryoconite on Flade Isblink are also highly variable (CV % > 75%). Until recently, despite being repeatedly observed, the intraglacial heterogeneity of radioactivity in cryoconite had not been assessed in detail. Due to the number of samples considered here (46 compared to typically <10 samples per location), it has been possible to explore such variability, suggesting that macroscopic features of cryoconite and the degree of maturity play an important role in the accumulation of atmospherically derived contaminants. However, it is acknowledged that these samples do not represent the entire ice surface of Flade Isblink.

The radioactive signature of radioactivity in cryoconite from Flade Isblink was compatible with a dual source: global stratospheric fallout and weapons-grade fissile material from USSR tests conducted in Novaya Zemlya. The plutonium isotopic signature and the excess of ^207^Bi support this view. They also confirm the hypothesis advanced decades ago^36^ that high-yield multimegaton atmospheric thermonuclear tests in Novaya Zemlya spread this radionuclide across the Arctic. Our results strengthen this and support the application of ^207^Bi as a marker for those specific events, allowing the refinement of sediment chronology for the 20th century, at least in the Arctic.

This study demonstrates that the surface of glaciers are highly dynamic environments, where the interaction between atmospheric deposition, meltwater and cryoconite translates into biogeochemical processes whose environmental implications are far from understood. It is unclear if radioactivity in cryoconite is increasing over time, but as interaction with meltwater is a key mechanism in the accumulation of FRNs, increased melt rates in response to climatic warming are likely to enhance the mobilization of FRNs in supraglacial environments. Furthermore, research has shown that FRNs attached to cryoconite are solubilized and mobile to different degrees,^72,73^ which may impact the secondary release of these FRNs back into meltwater and, ultimately, downstream environments. The low concentration of FRNs in sediments from a local stream fed by Flade Isblink suggests that cryoconite-related radioactivity is efficiently diluted after being released from the ice cap. However, this does not rule out the possibility that cryoconite accumulates in the fragile and transient environment linking the glacier to the surrounding proglacial terrain. Addressing these research gaps will advance our knowledge of FRN behavior in glacial environments, contributing to better radiological assessments and environmental management strategies in the changing Arctic.

## Supporting Information Available

The full data set used for this work is available on Pangea (https://doi.pangaea.de/10.1594/PANGAEA.966669). The Supporting Information document for this manuscript contains additional analytical details, cryoconite characteristics (Table S1), median data for FRN activities (Table S2), correlation matrix (Table S3), Quantile–Quantile plot of variables (Figure S1), variables compared by macroscopic features of cryoconite (Figure S2), comparison of variables to river sediments (Figure S3), and grain size distributions (Figure S4) (PDF)
