# Supplementary material for: Accumulation of Environmental Radioactivity on the Surface of a High Arctic Ice Cap (Flade Isblink, NE Greenland)
Source: Environ Sci Technol. 2024 Aug 6;58(38):17004–14. doi: 10.1021/acs.est.3c10755 (PMC11428132; doi:10.1021/acs.est.3c10755)
Supplement: Supplementary file 1 — es3c10755_si_001.pdf [file es3c10755_si_001.pdf]

Supporting information to: "Accumulation of environmental radioactivity on the surface of a High Arctic ice cap (Flade Isblink, NE Greenland)."

Dylan B. Beard[1*$], Giovanni Baccolo[2,3*$#], Caroline C. Clason[4], Geoffrey E. Millward[1], Edyta Łokas[5], Elena Di Stefano[6], Sally Rangecroft[7], Dariusz Sala[5], Przemysław Wachniew[8], William H. Blake[1]

[1]School of Geography, Earth and Environmental Sciences, University of Plymouth, PL4 8AA, UK
[2]Laboratory of Environmental Chemistry, Paul Scherrer Institut, Villigen, 5232, Switzerland
[3]Oeschger Centre for Climate Change Research, University of Bern, Bern, 3012, Switzerland
[4]Department of Geography, Durham University, Durham, DH1 3LE, UK
[5]Institute of Nuclear Physics Polish Academy of Sciences, 31-342 Kraków, Poland
[6]Physics Department, University of Milano-Bicocca, Milano, 20126, Italy
[7]School of Geography, University of Exeter, Exeter, EX4 4RJ, UK
[8]Faculty of Physics and Applied Computer Science, AGH University of Science and Technology, 30-059 Kraków, Poland

*Correspondence emails: dylan.beard@plymouth.ac.uk, giovanni.baccolo@uniroma3.it
$D.B.B. and G.B. contributed equally to this paper.

Number of pages: 12
Number of tables: 3
Number of figures: 4

Contents

1. Analytical details 3

1.1. Sample collection and treatment 3

1.2. Gamma spectrometry 3

1.3. Alpha spectrometry 3

1.4. Grain size analysis 4

1.5. Correlation study 4

2. Tables 5

Table S1. Characteristics used to classify Flade Isblink cryoconite samples (n=46). 5

Table S2. Median data for FRN activity concentrations determined in cryoconite and local riverine sediments at Flade Isblink. 6

Table S3. Correlation matrix (Pearson's correlation) calculated for the Flade Isblink (FI) cryoconite dataset. 7

3. Figures 8

Figure S1. Quantile-Quantile plot for the variables considered in this study. 8

Figure S2. Measured variables compared by macroscopic features of cryoconite. 9

Figure S3. Riverine sediments collected near FI compared to cryoconite sampled on FI (average values). 10

Figure S4. Particle size distribution of the samples considered for granulometric measurements. 11

4. References 12

1. Analytical details

1.1. Sample collection and treatment

Each sample was collected using a spoon and stored in clean 50ml plastic centrifuge tubes. Sampling sites were each photographed and described according to the size of the cryoconite deposit, its connection with meltwater channels and its aggregation state. Samples were successively dried at 50°C for 48 hours and transported to the University of Plymouth, UK, where gamma spectrometry was carried out. An aliquot of each sample was sent to the Institute of Nuclear Physics at the Polish Academy of Sciences for alpha spectrometry. All material was ground and sieved to <63µm using a ceramic mortar and pestle to ensure sample homogeneity. Each sample's loss on ignition was measured as a percentage weight loss through combustion at 600°C for six hours. The material was burned again at 950°C for two hours, cooled in a desiccator and re-weighed to determine the mass of the lost carbonate content.

1.2. Gamma spectrometry

The energy calibration of the gamma-detector (a well model, ORTEC GWL-170-15 S; N-type) was performed using soil samples spiked with a standard, calibrated, and certified solution (80717-669, Eckert & Ziegler Analytics, Georgia, USA), containing 12 radionuclides whose gamma emissions cover the gamma energy range from 46 keV to 1836 keV. The following gamma-emitting radionuclides were analyzed: ^{210}Pb (half-life ($t_{½}$) = 22.3 yr), ^{241}Am ($t_{½}$ = 432.2 yr), ^{214}Pb ($t_{½}$ = 26.8 min), ^{137}Cs ($t_{½}$ = 30.2 yr), ^{40}K ($t_{½}$ = $1.25 \cdot 10^9$ yr), ^{7}Be ($t_{½}$ = 53.3 d), ^{228}Ac ($t_{½}$ = 6 hr), and ^{207}Bi ($t_{½}$ = 32.9 yr). For each one of the listed radionuclides, the following gamma emissions were used to quantify their activity concentration: 46.52 keV, 59.54 keV, 295.34 keV (and 351.99 keV), 661.6 keV, 1460.8 keV, 477.6 keV, 911.1 keV (and 18 other lines), and 569.7 keV (and six other lines), respectively. The detector's performance was checked through quality control analyses of the IAEA moss soil IAEA-CU-2009-03.

1.3. Alpha spectrometry

Plutonium was separated from the sample matrix through acid digestion and radiochemistry. Samples were initially ashed at 600 °C and then mineralised with an acid mixture (HF, HNO_3, HCl and H_3BO_3). ^{242}Pu (NIST Standard Reference Materials 4334) was spiked to the samples to evaluate the recovery factor of the procedure. Pu was separated using the Dowex-1 ionic exchange resin, from which Pu was eluted with 8 M HNO_3 after adjustment of the oxidation state at $^{+4}$ using hydrazine and $NaNO_2$ (see reference[1]). After separation, plutonium was precipitated to create alpha spectrometric sources through NdF_3 - micro co-precipitation[2]. Alpha spectrometry was carried out on precipitated samples using a Canberra PIPS detector with an active surface of 450 mm^2 and a Canberra

alpha spectrometer. After measurements, membrane filters with deposited Pu isotopes were dissolved using concentrated acids to measure the $^{240}Pu/^{239}Pu$ isotopic ratio. A mixture of HNO_3, H_3BO_3, HCl and $HClO_4$ was used. After dissolution, the Pu oxidation state was again adjusted to $^{+4}$. To separate Pu from U and Th traces, the TEVA resin (Triskem) was used. Thorium was removed with 9M HCl. The Pu fraction was eluted using 0.1M HCl and 0.1M HF. Traces of organic resin commonly present in the Pu eluate were eliminated with the aid of concentrated $HClO_4$ and HNO_3. Pu salts were re-dissolved in 2% HNO_3 and 0.25% HF. Isotopic ratios were measured with the Agilent 8900#100 triple quadrupole ICP-MS and an Aridus desolvating nebuliser. To reduce polyatomic interferences, Pu isotopes were measured in mass shift mode (+32 amu) using a reaction with O_2 in collision reaction cell (CRC). Instrumental mass fractionation was monitored using IRMM-290 standard (EC, JRC) with certified $^{239}Pu/^{242}Pu$ atom ratios (0.10136 ± 0.00010 mol/mol).

1.4. Grain size analysis

Granulometric analyses were conducted on a subset of ten samples, including examples of all the types of collected cryoconite (wet/dry and fine/intermediate/granular). Analyses were performed through the Coulter counter technique. This is a technique relying on the principle that the electrical resistance of a conducting liquid is modified by the presence of insulating particles, and is a reference method for the analyses of fine mineral particles[3]. A Beckman Multisizer 4 was used for this purpose. The instrument was equipped with a 100 µm orifice, allowing measurement of particles with a spherical equivalent diameter between 2 and 60 µm, divided into 400 size channels. Samples were dried and gently worked with a mortar to break the cryoconite granules present, which was especially important for the granular cryoconite type. After preparation, an aliquot of about 0.5 g was added into 10 mL of ultrapure water (MilliQ system), with ultra-pure NaCl solution added until a final Na^+ concentration of 1% m/m was reached. The samples were agitated for a few seconds before measurement. For each sample 5 runs were performed, and for each run 0.5 mL were analysed. The runs took 15 seconds each and in between the runs the samples were shaken again to limit the settling effect of the larger particles as much as possible.

1.5. Correlation study

Multi-Dimensional Scaling (MDS) was used to graphically display the degree of correlation among the considered variables (radionuclides, LOI and carbonate content), using a similarity metric derived from the Pearson's correlation matrix[4]. The subset of samples for which Pu data were available (26 out of 48 samples) was considered. Hierarchical clustering was then applied to identify potential clusters revealed by MDS.

2. Tables

Table S1. Characteristics used to classify Flade Isblink cryoconite samples (n=46). Each sample was characterised by interaction with meltwater (wet or dry) as well as the aggregation state of the deposit (fin, intermediate, or granular).

Feature	Definition	Samples collected
Wet cryoconite	Material at least 80% submerged in water	26
Dry cryoconite	Material less than 10% submerged in water	20
Fine cryoconite	Cryoconite consisting of almost all fine material, with few or no granules	17
Intermediate cryoconite	Cryoconite containing mostly fine material but with some small granules (up to 40%)	20
Granular cryoconite	Cryoconite predominantly consisting of well-defined granules with very little or no fine material	9

Table S2. Median data for FRN activity concentrations determined in cryoconite and local riverine sediments at Flade Isblink. Median data is categorised according to aggregation state (fine, intermediate and granular) and interaction with supraglacial meltwater (wet or dry). The median for all cryoconite (cryoc.) samples is shown in the second column for comparison. The ± notation for river sediment values refers to measurement uncertainties of single samples (2-sigma). For the calculation of median values, data below MDA were replaced with MDA/2. Raw data can be found on Pangea at (https://doi.pangaea.de/10.1594/PANGAEA.966669).

Variable	All cryoc.	Fine cryoc.	Inter. cryoc.	Granular cryoc.	Wet cryoc.	Dry cryoc.	River-g	River-ng
**LOI (%)**	**3.9**	3.8	3.8	4.8	3.9	3.9	2.2	6.6
**Carbonate (%)**	**4.5**	4.5	4.3	5.4	4.5	4.5	2.8	12
**^{137}Cs (Bq kg^{-1})**	**219.3**	214	180	820	240	215	< 1.8	5.4±1.2
**^{207}Bi (Bq kg^{-1})**	**0.9**	0.9	0.9	1.0	0.9	0.9	< 0.7	< 0.7
**^{238}Pu (Bq kg^{-1})**	**0.3**	0.1	0.3	0.7	0.4	0.3	< 0.02	0.02±0.02
**^{239,240}Pu (Bq kg^{-1})**	**4.9**	3.4	4.3	15	5.2	4.7	< 0.02	0.31±0.06
**^{241}Am (Bq kg^{-1})**	**2.6**	2.6	2.3	11	2.5	2.8	< 0.7	< 0.7
**^{7}Be (Bq kg^{-1})**	**140.5**	270	51	75	240	100	< 11	90±30
**^{210}Pb$_{exc.}$ (Bq kg^{-1})**	**712.3**	760	670	2220	800	710	10±20	35±25
**^{40}K (Bq kg^{-1})**	**1836**	1840	590	2040	1140	1900	1680±210	1200±150
**^{228}Ac (Bq kg^{-1})**	**92.4**	84	94	96	93	90	66±14	63±13
**^{214}Pb (Bq kg^{-1})**	**30.9**	31.1	30.5	32.2	30.8	31.3	23±5	32±6

Table S3. Correlation matrix (Pearson's correlation) calculated for the Flade Isblink (FI) cryoconite dataset. Correlation coefficients are only shown for variables above 95% significance ($p<0.05$).

	**LOI (%)**	**Carbonate (%)**	**^{137}Cs**	**^{214}Pb**	**^{210}Pb$_{exc.}$**	**^{241}Am**	**^{207}Bi**	**^{228}Ac**	**^{7}Be**	**^{40}K**
**LOI (%)**	1.00	0.90		0.53						
**Carbonate (%)**	0.90	1.00		0.39						
**^{137}Cs**			1.00		0.90	0.98	0.57			
**^{214}Pb**	0.53	0.39		1.00				0.30		
**^{210}Pb$_{exc.}$**			0.90		1.00	0.86	0.44	0.30		
**^{241}Am**			0.98		0.86	1.00	0.57			
**^{207}Bi**			0.57		0.44	0.57	1.00			
**^{228}Ac**				0.30	0.30			1.00		
**^{7}Be**									1.00	
**^{40}K**										1.00

0.8 - 0.1
0.6 - 0.8
0.4 - 0.6
0.2 - 0.4
0.0 - 0.2
-0.2 - -0.0
-0.4 - -0.2
-0.4 - -0.6
-0.6 - -0.8
-0.8 - -0.0

3. Figures

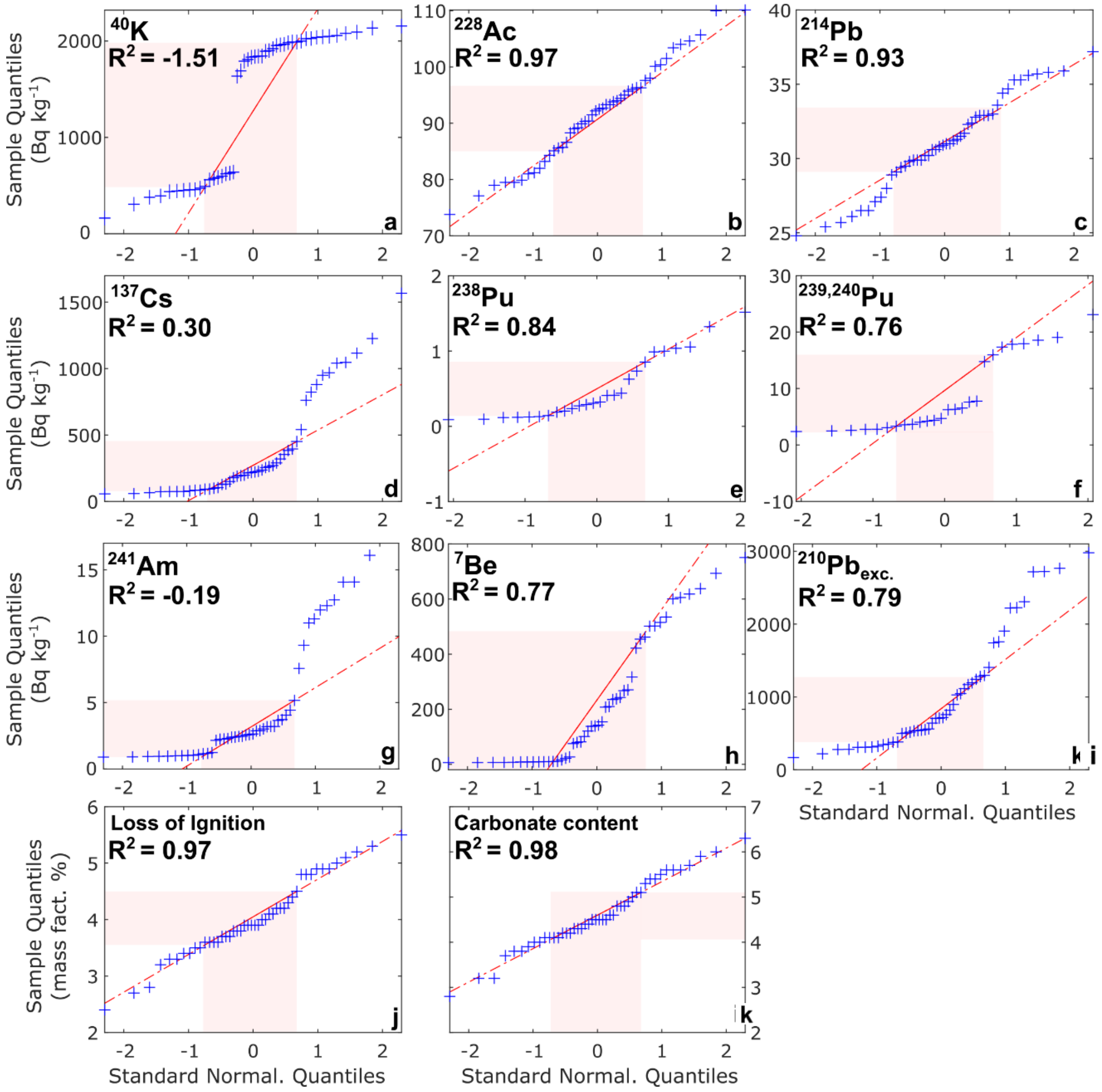

Figure S1. Quantile-Quantile plot for the variables considered in this study. Geogenic radionuclides (panels a-c); artificial fallout radionuclides (panels d-g); natural fallout radionuclides (panels h, i); loss on ignition and carbonate content (panels j, k). Red areas mark the interval between the first and third quantiles of data distributions. Variables displaying a well-represented distribution by the red normality line ($R^2 > 0.8$) were considered normally distributed, including ^{228}Ac, ^{214}Pb, ^{238}Pu, LOI, and carbonate content.

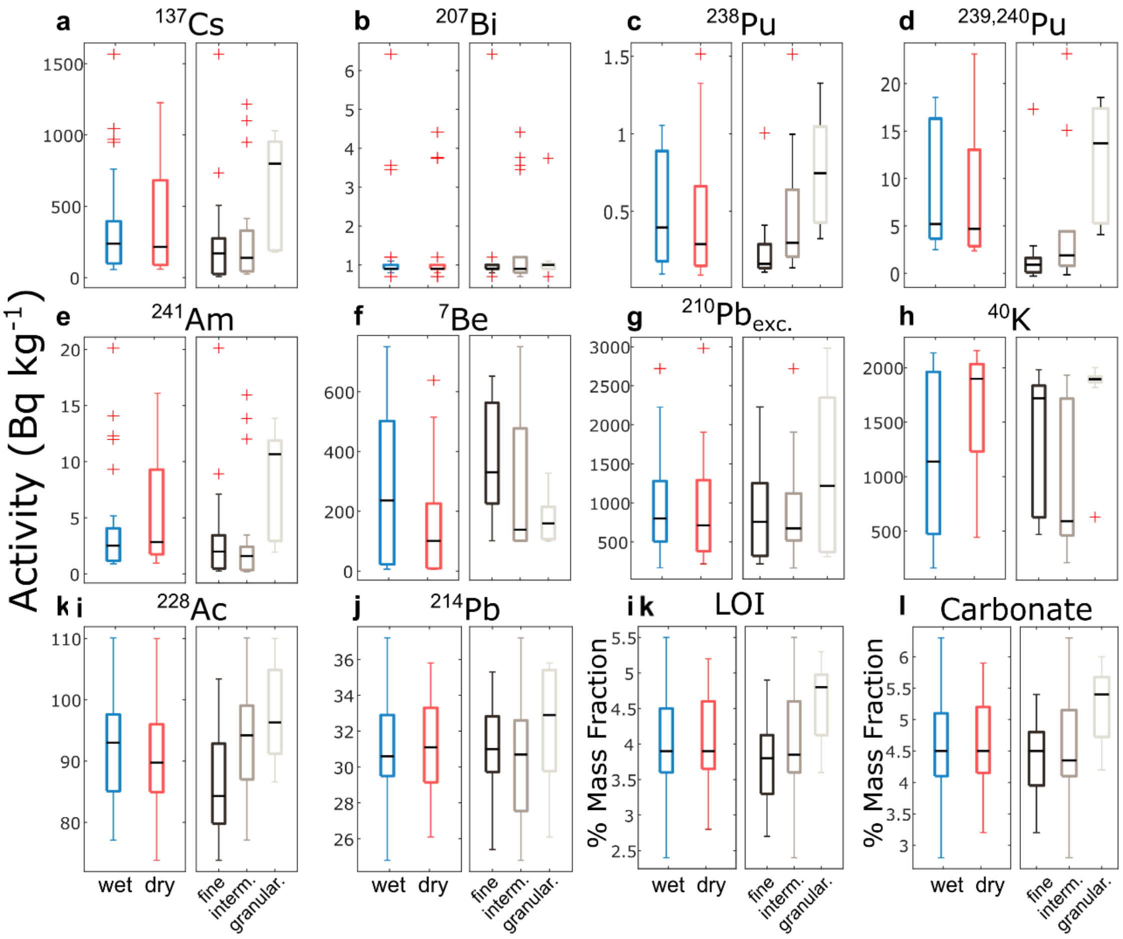

Figure S2. Measured variables compared by macroscopic features of cryoconite. Radioactivity (panels a-j), LOI (panel k) and carbonate content (panel l) in FI cryoconite samples were divided according to their degree of interaction with meltwater (wet and dry, the first graph of each pair) and to their aggregation state (fine, intermediate and granular, the second graph of each pair).

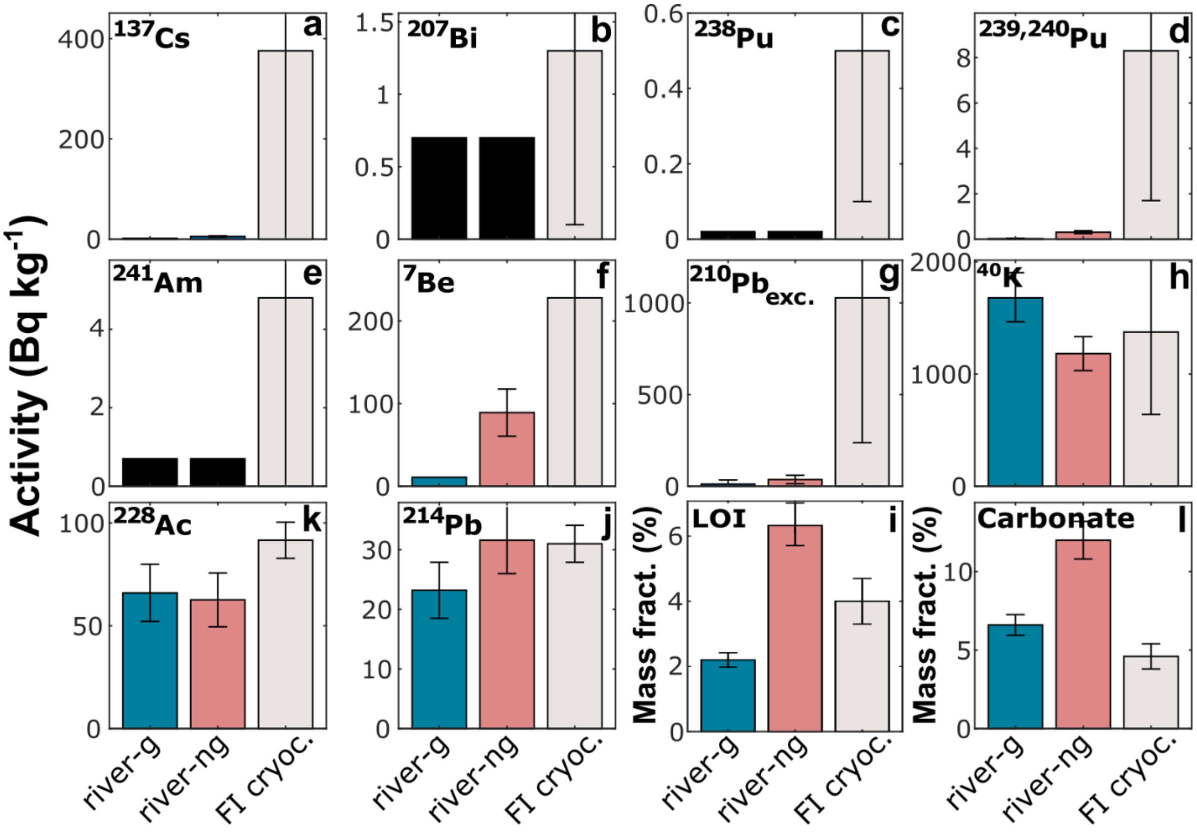

Figure S3. Riverine sediments collected near FI compared to cryoconite sampled on FI (average values). Black bars refer to MDA for activities below the detection limit. "*river-g"* refers to sediments collected from the main glacial river of Flade Isblink in the Prinsesse Ingeborg Halvø; "*river-ng"* refers to channel bank deposits from a river fed by snowmelt; "FI cryoc." refers to cryoconite from Flade Isblink. Error bars refer to the experimental uncertainty of the river sediments and one standard deviation for cryoconite.

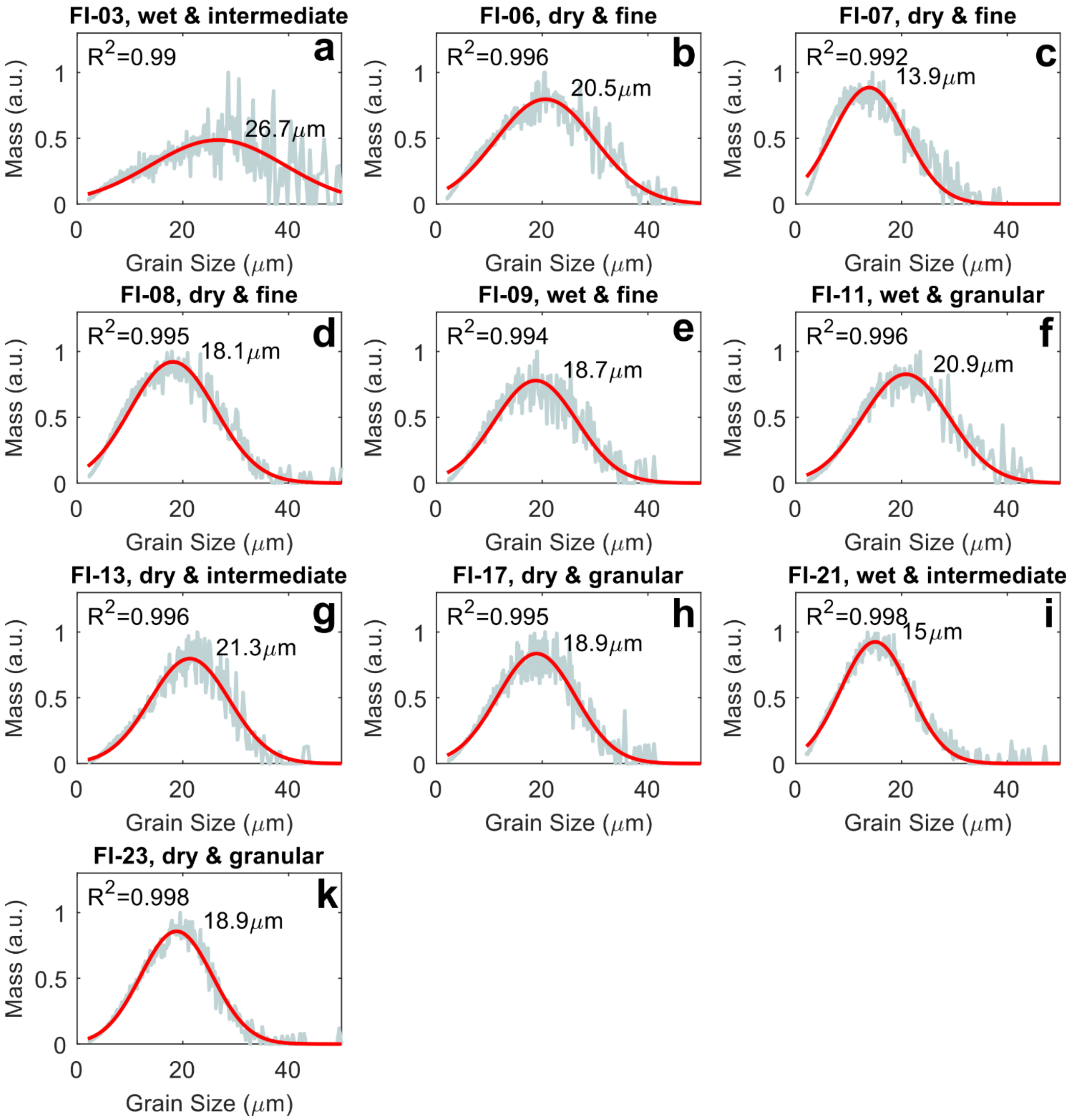

Figure S4. Particle size distribution of the samples considered for granulometric measurements. The grey curve refers to raw instrumental data, and the red curve to a gaussian fit applied to samples. For each panel, the coefficient of determination (R^2) and the mode are reported. Data are expressed as arbitrary units, where 1 the highest particle size concentration observed in each distribution.

4. References

(1) LaRosa, J. J.; Cooper, E. L.; Ghods-Esphahani, A.; Jansta, V.; Makarewicz, M.; Shawky, S.; Vajda, N. Radiochemical Methods Used by the IAEA's Laboratories at Seibersdorf for the Determination of 90Sr, 144Ce and Pu Radionuclides in Environmental Samples Collected for the International Chernobyl Project. *Journal of Environmental Radioactivity* **1992**, *17* (2), 183–209. https://doi.org/10.1016/0265-931X(92)90025-O.

(2) Łokas, E.; Zaborska, A.; Kolicka, M.; Różycki, M.; Zawierucha, K. Accumulation of Atmospheric Radionuclides and Heavy Metals in Cryoconite Holes on an Arctic Glacier. *Chemosphere* **2016**, *160*, 162–172. https://doi.org/10.1016/j.chemosphere.2016.06.051.

(3) Ruth, U.; Barbante, C.; Bigler, M.; Delmonte, B.; Fischer, H.; Gabrielli, P.; Gaspari, V.; Kaufmann, P.; Lambert, F.; Maggi, V.; Marino, F.; Petit, J.-R.; Udisti, R.; Wagenbach, D.; Wegner, A.; Wolff, E. W. Proxies and Measurement Techniques for Mineral Dust in Antarctic Ice Cores. *Environ. Sci. Technol.* **2008**, *42* (15), 5675–5681. https://doi.org/10.1021/es703078z.

(4) Baccolo, G.; Łokas, E.; Gaca, P.; Massabò, D.; Ambrosini, R.; Azzoni, R. S.; Clason, C.; Di Mauro, B.; Franzetti, A.; Nastasi, M.; Prata, M.; Prati, P.; Previtali, E.; Delmonte, B.; Maggi, V. Cryoconite: An Efficient Accumulator of Radioactive Fallout in Glacial Environments. *The Cryosphere* **2020**, *14* (2), 657–672. https://doi.org/10.5194/tc-14-657-2020.
